# Construction Notes

**Published:** 1894-05-19

**Authors:** 


### CONSTRUCTION NOTES.

### Infectious Diseases Hospital, Birkenhead.

The foundation-stone of the above has just been laid. The site of the hospital contains an area of about 5½ acres, and is nearly two miles from the Town Hall. The building as arranged at present to be erected is the administrative block, isolation or private ward pavilions, laundry and disinfector block, mortuary and van-house, giving accommodation for 44 beds; but there is room on the site to erect five additional ward pavilions, giving a total of 104 beds. Each of the pavilions and the administrative block have their fronts to the south. The buildings are all of a plain but substantial character, of local grey bricks, with Ruabon-pressed brick strings and dressings. The floors of all the wards are to be pitch-pine, thoroughly ventilated underneath by a continuous course of perforated blue bricks, which in addition to ensuring a thorough cross ventilation under the joists and floors, will also act as a second damp-proof course. Messrs. Kelly Brothers, of Liverpool contracted for the building, and Mr. Charles Brownridge, C.E., Borough Surveyor of Birkenhead, prepared the architectural designs.

### Infectious Diseases Hospital, Stratford-on-Avon.

The ceremony of laying the foundation-stone of the joint Infectious Diseases Hospital about to be erected at Stratford-on-Avon, at the expense of the urban and rural sanitary authorities was performed early in this month. The hospital is to be erected on a site belonging to the Corporation, and situated about a mile from the town. The plans were prepared by the borough surveyor, Mr. A. H. Campbell. There will be five blocks, including the administrative departments all detached. The wards are on the pavilion system, and are designed with a view to enlargement if necessary. The administrative block provides accommodation for the matron and staff of nurses. The disinfecting chamber will be furnished with a Washington Lyon steam disinfector. The buildings will all be of brick, with stone dressing, and their erection has been entrusted to Mr. G. Whateley, a local tradesman. The combined district to be served by the hospital contains a population of about 22,000, and the building has accommodation for 20 beds. The estimated cost, including the furnishing, building, and drainage is £5,872, which will be borne three-fifths by the rural, and two fifths by the urban authority.

### New Hospital at Thurso.

The architect of this hospital is Mr. Cronin, C.E., of Wick. The rooms in connection with the management of the hospital are in the centre, and the wards, which are three, are to the right and the left and rear of the centre block. These are connected by lofty corridors running behind the administrative block. There are two small observation wards, which will be used for doubtful cases or for the convenience of nursing. The number of beds arranged for is 20, but the buildings are constucted for further extension. The administrative block is large and commodious, so that it is sufficient for further increase of ward accommodation. The outbuildings are to be erected at a convenient distance from the hospital, and will consist of washing-house, laundry, morcuary, disinfecting chamber, coal-house, and ambulance-house. The bath-room and water-closet accommodation is placed at the end of each ward, and is separated therefrom by a small passage with cross ventilation. The walls of all the buildings are to be of native stone, with freestone facings. The floors will be of wood, and the areas under the floors will be covered with cement concrete. Wards, corridors, and bath-rooms will be ventilated with Boyle's exhaust ventilators and by Tobins' tubes. It is intended in the meantime to heat this building by open fires, but provision will be made for afterwards introducing heating by hot water or air should it be found necessary. An ample supply of excellent water is to be taken from springs in the neighbourhood, and the system of sewerage will be carried out with the latest improvements.

